# The combined impacts of toxic drug use and the 2021 Heat Dome in Canada: A thematic analysis of online news media articles

**DOI:** 10.1371/journal.pone.0318229

**Published:** 2025-01-31

**Authors:** Emily J. Tetzlaff, Nicholas Goulet, Melissa Gorman, Glen P. Kenny

**Affiliations:** 1 Human and Environmental Physiology Research Unit, School of Human Kinetics, University of Ottawa, Ottawa, ON, Canada; 2 Heat Division, Climate Change and Health Office, Healthy Environments and Consumer Safety Branch, Health Canada, Ottawa, ON, Canada; 3 Behavioural and Metabolic Research Unit, School of Human Kinetics, Faculty of Health Sciences, University of Ottawa, Ottawa, ON, Canada; 4 Clinical Epidemiology Program, Ottawa Hospital Research Institute, Ottawa, ON, Canada; Vancouver Coastal Health Research Institute, CANADA

## Abstract

**Introduction:**

During the summer of 2021, western Canada experienced a historic and deadly extreme heat event. Simultaneously, toxic drug use and overdoses related to high-risk use of opioids and polysubstance use continued to rise across the country. However, the combined impacts of these intersecting public health crises remain poorly understood as relevant data sources are limited in Canada.

**Methods:**

We explored news media articles (*n* = 86, 3%) discussing toxic drug use, overdose-related deaths and the 2021 Heat Dome which were identified in a systematic review of Canadian online news media (e.g., newspaper articles, radio broadcasts) from five subscription news databases and an extensive grey literature search (*n* = 2909). The analyzed articles were published before, during and after the 2021 Heat Dome, and were qualitatively coded and thematically analyzed in NVivo to identify patterns of meaning across the dataset.

**Results:**

Four main themes were identified within our media-based analysis: (I) the reported impact of toxic drug use on human thermoregulation and behavioural adaptation; (II) the reported demand of intersecting crises on the health system; (III) barriers and stigma reported to influence an individual’s access to or use of heat mitigation behaviours and services; and (IV) the reported impact of extreme heat on the public health response to drug poisoning emergencies.

**Conclusion:**

With increasing temperature extremes and a rising tide of toxic drug use and overdoses, our findings illustrate that there is a need for further research to better understand the combined impacts when toxic drug use, overdose-related deaths, and extreme heat coincide.

## Introduction

In the summer of 2021, western Canada experienced an unprecedented extreme heat event (EHE) (termed the ‘2021 Heat Dome’) that claimed the lives of 619 people in British Columbia (B.C.) [1], marking it one of the deadliest recorded natural disasters in Canadian history. Throughout the nearly 2-week EHE, more than 103 all-time heat records were broken, including Canada’s highest ever measured temperature (49.6°C Lytton, B.C. on June 29th, 2021), and the highest temperature ever recorded north of latitude 60° (39.9°C Fort Smith, Northwest Territories on June 30th, 2021) [1]. Simultaneously, toxic drug use and overdoses related to high-risk use of opioids and polysubstance use continued to rise in Canada and were responsible for an estimated 5,368 deaths between January and September 2021, with most deaths (88%) occurring in British Columbia and Alberta [2]. The convergence of these events in the summer of 2021 in western Canada placed an unprecedented strain on individual and community health.

While some studies internationally have explored the intersection of toxic drug use, overdose-related deaths, and extreme heat [3, 4], these types of studies are scarce in Canada. As such, their specific juncture during the 2021 Heat Dome remains poorly understood. Improving our understanding of the combined impacts of toxic drug use and extreme heat in Canada is especially important as overdose and heat-related mortality are among the deadliest preventable health issues in Canada. Further, both toxic drug use and extreme heat can disproportionately impact specific and overlapping at-risk populations, such as individuals experiencing housing insecurity or individuals who live in unairconditioned environments. For example, there is evidence suggesting that drug use in hot settings, such as housing without air conditioning, parked cars or outdoors during EHEs increases heat exposure (i.e., the individual may experience sedative effects or loss of consciousness that make them unable to respond to their environment), leading to an increased risk of heat-related illnesses and death [3]. As EHEs are on the rise and will affect communities more frequently, severely and for longer periods, it is essential that we understand the relationship between harmful substance use and heat vulnerability. Yet, without an analysis of past events where toxic drug use, overdose-related deaths, and extreme heat coincided, it remains unclear how these factors could combine in the future to impact human health and health systems, and what public health interventions might be most effective.

Data sources for the combined impacts of toxic drug use, overdose-related deaths, and exposure to extreme heat are limited in Canada and globally [5], making it challenging to conduct analyses of past events. However, various recent studies have used online news media articles to capture information about the combined health impacts of two or more health crises, such as an EHE and the COVID-19 pandemic [6]. Notably, online news media articles serve as a significant source of information to the public [7] and can provide a unique medium for understanding the impacts of intersecting crises. The media can also capture stories of how people, organizations, infrastructure, and the economy are impacted since journalists draw on a wide array of sources and perspectives [8]. Further, with the public receiving a significant amount of their health information from the media, analyzing articles as a data source provides unique insight into the type of coverage and content being disseminated. Although several analyses of online news media articles of individual EHEs have been undertaken globally [8, 9], few have been conducted within the Canadian context [9]. To help fill these gaps and improve our understanding of the health and health system impacts of toxic drug use, overdose-related deaths, and extreme heat, we systematically reviewed and thematically analyzed online news media articles published before, during, and after the 2021 Heat Dome. For this analysis, we distinguish toxic drug use from the use of prescribed substances and drugs/medications used under medical supervision.

## Materials and methods

### Search strategy and article selection process

This study draws on a subset of data collected for an extensive analysis of online news media articles related to the 2021 Heat Dome in Canada [10]. The primary investigation included a systematic review of digitized media content (e.g., newspaper articles, radio broadcasts and television transcripts) from five subscription news databases (ProQuest Canadian Major Dailies, Business Source Elite, NewsDesk, Factiva, and Eureka). To minimize reliance on prestige press (e.g., The Globe and Mail, Toronto Star, The Province) and limit outlet bias, we used five subscription news databases (ProQuest Canadian Major Dailies, Business Source Elite, NewsDesk, Factiva and Eureka). These databases provide access to current content and significant backfiles for Canada’s highest-circulating national and regional newspapers and smaller outlets in full-text format. The search strategy was developed in consultation with a Research Librarian, and the final search underwent PRESS review before database translation (see S1 File). For all searches, the content was limited to English and French articles published within Canada between 1 June 2021 and 26 February 2022. This study excluded all social media posts (e.g., X [formerly Twitter] and Facebook) and content without verbatim transcription (e.g., audio and video-only content). To supplement the database searches, a list of targeted public and non-profit organization websites (n = 997) was created for each province and territory in Canada, along with pan-Canadian sites and an Advanced Google search for key terms (e.g., “heat” and “2021”) (see S1 File for more details).

The articles were uploaded to Zotero (Release 6.0, Corporation of Digital Scholarship), a digital citation tool. As some articles were released to multiple news sources (due to media conglomerations sharing and modifying content), similar versions of articles were identified, and deduplication was completed. French articles were identified and sent for professional translation. The translated documents were then checked for accuracy by a bilingual research team member. The articles were then reviewed to ensure that the data captured included only Canadian-produced news articles and that the text primarily focused on the Canadian context. The resulting dataset for the primary analysis included 2,909 articles (see S2 File for more details).

**Fig 1:**
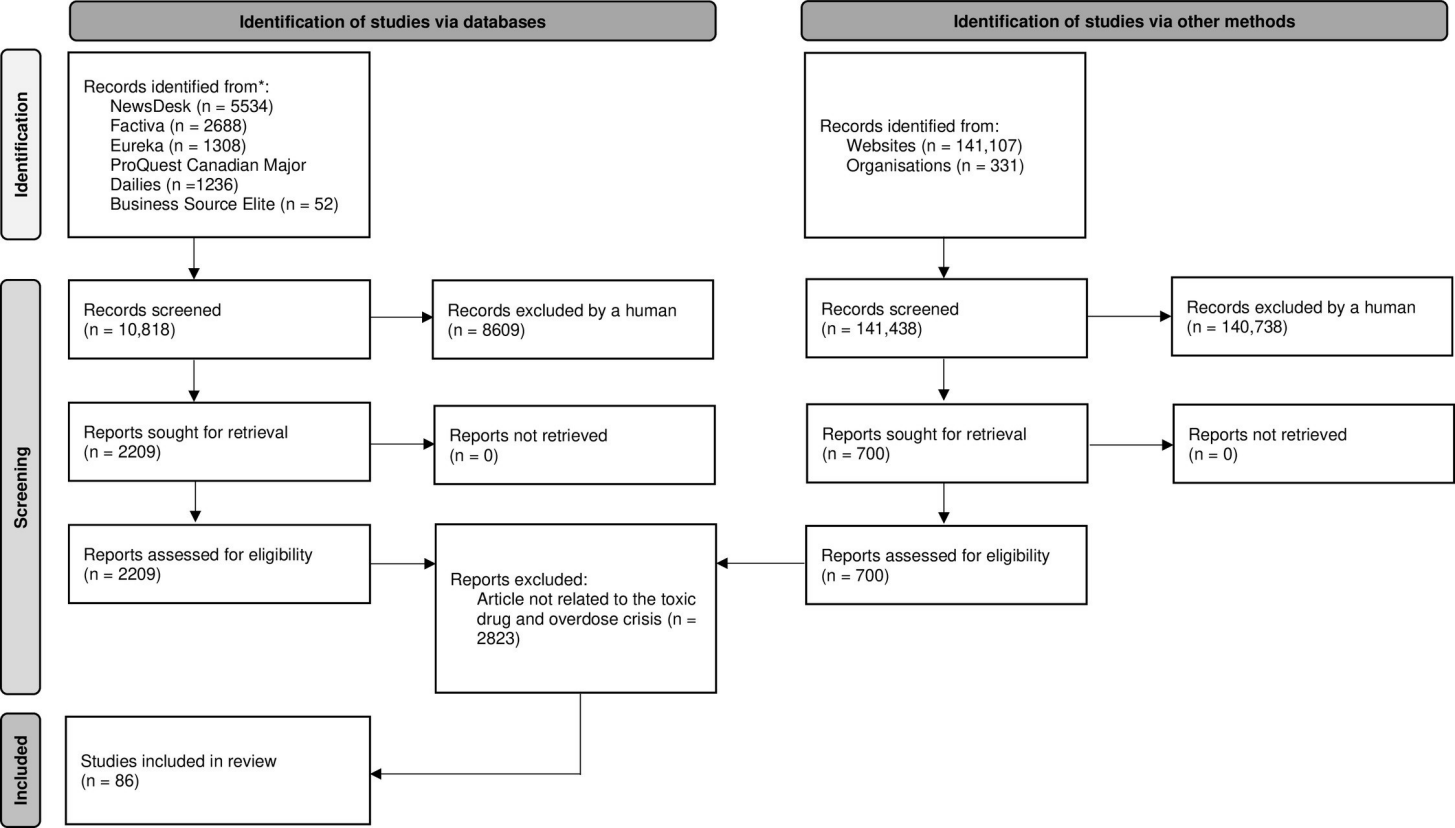
PRISMA-ScR flow diagram of search and study selection process. PRISMA-ScR: Preferred Reporting Items for Systematic Reviews and Meta-analyses for Scoping Reviews.

All articles (n = 2,909) were uploaded to NVivo, a qualitative content analysis software. The authors then developed a list of key terms to serve as indicators of content relevant to toxic drug use and overdose-related deaths (**Table 1**). The NVivo ‘Text Search’ function was then used to search each identified reference term within the article database (E.J.T.). The search identified 86 potential articles (**Table 1**: Reference Count). Two authors then independently reviewed the articles to confirm they met the inclusion criteria (E.J.T. and N.G.) (**Fig 1**) (see S2 File for more details).

**Table 1:** Terms used as positive indicators of relevant content.

Term	Reference Count (n)
**Overdose**(s) (Overdose alert, Overdose crisis, Overdose deaths)	56
**Drug**(s) (Drug toxicity deaths, Drug use, Illicit drug deaths, Illicit drug overdose, Illicit drug toxicity, Illicit drugs, Toxic drug crisis, Toxic drug supply, Toxic drugs, Toxic drug overdose, Local drug supply, Safe drug supply, People with drug use problems)	48
**Opioid**(s) (Opioid Crisis, Opioid drug overdoses, Opioid epidemic, Opioid health crisis, Opioid overdose crisis, Opioid overdose, Opioid-related death)	37
**Substance**(s) (Substance use, Substance use issues, Substances, Substances users, Clients who may be using substances, Inject substances, Residents that use substances)	26
**Addiction**(s) (Addiction problem, mental health and addictions, community members who are dealing with addiction, People with addictions)	9
**Naloxone** (Naloxone kit, Overdose-reversing naloxone)	7
**Amphetamines**	4
**Fentanyl**	4
**MDMA** (or ecstasy)	4
**Cocaine**	3
**Cannabis**	2
**Crystal meth**	2
‘**Fire’** *	2
**Methamphetamine**(s)	2
**Heroin**	1
**LSD** (Lysergic acid diethylamide)	1
**Muscle relaxants**	1
**Painkillers**	1
**PCP** (Phencyclidine hydrochloride)	1

**Note:** The bolded terms represent the root word searched using the NVIVO ‘Explore > Text Search’ function. The search was conducted to include stemmed/root words. The bracketed terms then represent examples of terms that were identified in-text related to the root word.

* A dark purple highly toxic substance sold as ‘down or fentanyl.’

### Data analysis

The identified media articles were then analyzed using thematic analysis, a method that offers a way of identifying repeated patterns of meaning across a dataset [11]. The study was theoretically underpinned by constructivism, with an inductive orientation allowing for the themes identified to be strongly linked to the data. A semantic approach was also employed, meaning that the identified themes are based on the explicit or surface meaning of the data [12]. This approach aligned with this study’s media-based dataset as it emphasizes the lens of the media makers (e.g., journalists) in the initial interpretation of what is reported to the public [13]. Correspondingly, the analysis used a primarily descriptive form of thematic analysis that prioritized how the media presented meanings and experiences in developing the themes [14]. This method is particularly useful when investigating an under-researched area [14].

To conduct the thematic analysis, this study followed the six steps outlined by our colleagues [11] using NVivo, a qualitative content analysis software. The two primary coders initially familiarized themselves with the data during the primary project coding; however, they then re-immersed themselves via repeated active reading (i.e., reading while searching for meanings and patterns) of the smaller subset of articles (*n* = 86) of interest to this secondary analysis. Once the primary coders felt they had a strong comprehension of the breadth of the dataset, they then transitioned to the generation of initial codes from the data, where codes refer to the most basic segment, or element, of the raw data or information that can be assessed in a meaningful way regarding the phenomenon [15]. Next, themes were identified by sorting the identified codes (*n* = 13) and collating relevant data references [11]. During the analysis, coded extracts of data were allowed to be assigned to as many different themes as they fit into. The themes were then refined and defined by rereading all the collated extracts for each theme and considering whether they appeared to form a coherent pattern in relation to relevant literature. All authors then considered the validity of each individual theme in relation to the dataset and whether it accurately reflected the meanings evident in the dataset as a whole [11].

## Results

Four main themes were identified within the dataset: (I) the reported impact of toxic drug use on human thermoregulation and behavioural adaptation; (II) the reported demand of intersecting crises on emergency healthcare services; (III) barriers and stigma reported to influence an individual’s access to or use of heat mitigation behaviours and services; and (IV) the reported impact of extreme heat on the public health response to drug poisoning emergencies.

### Theme I: The reported impact of toxic drug use on thermoregulation and behavioural adaptation

The reported impact of toxic drug use on thermoregulation and heat mitigation behaviours was the most cited concern within the captured news media articles (**Table 2**). Although most of the articles did not provide physiological explanations, references to thermoregulation being compromised were identified. For example, a physician was quoted saying *“substance users are really at high risk…Their thermoregulatory systems just don’t work as well and if they’re impaired at all and don’t recognize the early warning signs*, *they can get very sick”* (Ref. 1). Often, these articles noted that individuals are at greater risk of heatstroke, dehydration, and other heat-related illnesses due to toxic drug use compromising their thirst sensation and impairing their ability to self-identify signs and symptoms. For example, one article quoted information from a provincial public health authority: *“alcohol and illegal drugs such as methamphetamines*, *amphetamines*, *cocaine*, *heroin*, *PCP (phencyclidine hydrochloride)*, *and LSD (lysergic acid diethylamide) can also cause problems*. *They affect how well you can sense dehydration or heat-related symptoms*. *They also make you less able to judge whether you need treatment for a heat-related illness”* (Ref. 2).

**Table 2:** Themes and example quotes from online news media articles discussing the intersection of toxic drug use, overdose-related deaths, and the 2021 Heat Dome in Canada.

Theme	Example Quote
**Theme I: The reported impact of substance use on thermoregulation and behavioural adaptation** (n = 41)	• *“Substance users are really at high risk…Their thermoregulatory systems just don’t work as well and if they’re impaired at all and don’t recognize the early warning signs*, *they can get very sick”* (Ref. 1)
	• *“Alcohol and illegal drugs such as methamphetamines*, *amphetamines*, *cocaine*, *heroin*, *PCP (phencyclidine hydrochloride)*, *and LSD (lysergic acid diethylamide) can also cause problems*. *They affect how well you can sense dehydration or heat-related symptoms*. *They also make you less able to judge whether you need treatment for a heat-related illness”* (Ref. 2)
	• “*MDMA can alter mental status as well as increase the risk of hyperthermia (over-heating)”* (Ref. 3)
	• *“Just with the dehydration factor*, *any substances that they’re using (have) a greater effect if their body isn’t in full defence mode”* (Ref. 4)
	• *“Combined with the heat*, *the new contamination in the local drug supply could be deadly*. *If an individual is unconscious for an hour or two because they’re heavily sedated*, *the effects of that kind of heat could be fatal*, *very quickly”* (Ref. 5)
	• “*Further compounding risks of extreme heat for people who are experiencing homelessness is drug use*, *which can exacerbate the symptoms of heat exhaustion or lead to secondary issues… When you’re using substances*, *you’re just going to have more severe side effects*. *People who are using methamphetamine and crystal meth are more at risk of heat stroke usually*. *People who are very dehydrated*, *it’s very hard to inject substances and you’re more prone to needle injuries and stuff like that”* (Ref. 6)
	• *“The delayed reactions when you drink alcohol or take drugs–including painkillers and muscle relaxants–can increase risk of an accident*. *Your reaction time*, *your depth perception*, *your reasoning*, *all of those things are diminished…So anything you can do to keep your wits about you and keep your mind functioning and your body in optimal shape when you’re near the water is important”* (Ref. 7)
**Theme II: The reported demand of intersecting crises on emergency healthcare services** (n = 31)	• *“The culmination of higher call volumes*, *the COVID-19 pandemic*, *extreme weather events*, *and the worsening overdose crisis make the job more physically and mentally taxing for paramedics”* (Ref. 8)
	• *“Emergency calls had increased dramatically during a record-breaking heat wave this summer that killed almost 600 people when paramedics were already under pressure from the overdose crisis”* (Ref. 9)
	• *“Ambulance-paramedic services countrywide are reporting more calls and fewer staff because of the nearly two-year-old COVID-19 pandemic and the ongoing drug overdose crisis*. *He adds extreme weather events*, *like last summer’s deadly heat wave in B*.*C*., *have highlighted the need for more staff and better mental health supports for first responders”* (Ref. 10)
	• *“365 calls*, *including cardiac emergencies*, *heat emergencies and overdoses…a call volume spike [of] three-fold compared to earlier [in] 2021”* (Ref. 11)
	• *“Chief Coroner Lisa Lapointe confirmed a ‘sharp’ increase in reported deaths have been driven by the opioid crisis and the summer’ heat dome’ event […]*, *which she said has ‘challenged existing resources’”* (Ref. 12)
	• *“The tally*, *four times the average number of fatalities*, *overwhelm[ed] coroners already overburdened by the toxic drug crisis…we are experiencing stress among the coroners*, *probably more than we’ve ever seen”* (Ref. 13)
**Theme III: The barriers and stigma reported to influence an individual’s access to or use of heat mitigation behaviour and services** (n = 8)	• “I’m *a drug addict*. *It’s hard going to just any old place because people are judgmental… I’d prefer to be outside* …*somewhere with big shady trees”* (Ref. 14)
	• *“[City Council] learned that homeless people cope with unusual heat with minimal protection*, *often have limited access to drinking water*, *and may face exclusion due to stigma from cooling off in air-conditioned spaces such as malls and other publicly accessible facilities”* (Ref. 15) • *“For those living on the street or who have severe mental-health or substance-use issues*, *the concern is even greater*. *Some of those individuals… won’t have access to shelter and other ways to keep cool*, *he said*” (Ref. 16)
**Theme IV: The reported impact of extreme heat on the public health response to drug poisoning emergencies** (n = 6)	• *“Many pharmacies received calls about drugs [i*.*e*., *Naloxone–an opioid antagonist] getting spoiled during the heat… naloxone kits are all required to be stored between 15°C and 25°C… naloxone kits are less effective when stored above recommended temperatures and may interfere with the drugs’ function*^”^ (Ref. 17)
	• *“The combined threats have [health and social service providers] scrambling to get ready at drop-in overdose prevention sites”* (Ref. 18)

**Note:** References to Theme I (*n* = 23), Theme III (*n* = 6), and Theme IV (*n* = 6) were more common during the 2021 Heat Dome (24 June 2021 to 7 July 2021) compared to after the event (*n* = 11, 0, and 2, respectively) (8 July 2021 to 26 February 2022). Theme II was more common after the 2021 Heat Dome (*n* = 25) compared to during the event (*n* = 6).

Many articles described how health outcomes are compounded using toxic drugs such as opioids in combination with stimulants (e.g., cocaine, methamphetamines) and other substances (termed ‘polysubstance use’). For example, one article cited a provincial health authority and stated that MDMA *“can alter mental status as well as increase the risk of hyperthermia (over-heating)”* (Ref. 3), by increasing metabolism, which results in greater heat generation while suppressing thirst. Various health professionals elaborated that *“just with the dehydration factor*, *any substances that they’re using (have) a greater effect if their body isn’t in full defence mode”* (Ref. 4), meaning that exposure to extreme heat may exacerbate the effect of the toxic drugs. Further, once experiencing the effects of toxic drugs, it was reported that the *“capacity for selfcare”* (Ref. 4) and ability to respond to the heat may become further impaired and can become fatal. For example, a representative from a drop-in overdose prevention site said: *“Combined with the heat*, *the new contamination in the local drug supply could be deadly*. *If an individual is unconscious for an hour or two because they’re heavily sedated*, *the effects of that kind of heat could be fatal*, *very quickly”* (Ref. 5). A similar sentiment was shared by the director of an outreach and clinical support service provider, stating *“the dangers associated with substance use may [further] be amplified”* and that many of these individuals *“won’t have access to shelter and other ways to keep cool”* (Ref. 6).

Some articles emphasized specific concerns for certain toxic drugs. For example, a coordinator with an integrated care centre said *“Further compounding risks of extreme heat for people who are experiencing homelessness is drug use*, *which can exacerbate the symptoms of heat exhaustion or lead to secondary issues… When you’re using substances*, *you’re just going to have more severe side effects*. *People who are using methamphetamine and crystal meth are more at risk of heat stroke usually*. *People who are very dehydrated*, *it’s very hard to inject substances and you’re more prone to needle injuries and stuff like that”* (Ref. 7). A subset of the articles also discussed using water immersion (e.g., swimming) as a heat mitigation strategy, but included additional public safety messaging to ensure toxic drugs and alcohol are avoided. For example, one article quoted a member of the Royal Canadian Mounted Police saying *“The delayed reactions when you drink alcohol or take drugs–including painkillers and muscle relaxants–can increase risk of an accident*. *Your reaction time*, *your depth perception*, *your reasoning*, *all of those things are diminished…So anything you can do to keep your wits about you and keep your mind functioning and your body in optimal shape when you’re near the water is important”* (Ref. 8).

### Theme II: The demand of intersecting crises on emergency healthcare services

As a result of the intersecting crises, various segments of the emergency healthcare system in western Canada were reported to have been impacted, including pre-hospital emergency services (e.g., paramedic services, dispatch, fire service), the provincial coroner’s service, and hospital services. Based on the captured news media articles, the sector most reported on by the simultaneous crises was the paramedic services which faced *“the culmination of higher call volumes…extreme weather events*, *and the worsening overdose crisis ma[king] the job more physically and mentally taxing for paramedics”* (Ref. 9). Numerous articles emphasized paramedics being *“stretched to their limits during the heat wave”* (Ref. 10), including frequent citations from the B.C. Minister of Health who stated that *“calls had increased dramatically during a record-breaking heat wave this summer that killed almost 600 people when paramedics were already under pressure from the overdose crisis”* (Ref 11). The President of the Paramedic Association of Canada was also quoted saying that the *“ambulance-paramedic services countrywide are reporting more calls and fewer staff because of…the ongoing drug overdose crisis*. *He adds extreme weather events*, *like last summer’s deadly heat wave in B*.*C*., *have highlighted the need for more staff and better mental health supports for first responders”* (Ref. 12). Similarly, based on the task organization of the emergency services in British Columbia, the dispatchers (E-Comm 911) were also impacted by the increased demand as they *“handle ninety-nine percent of all emergency calls in the province”* (Ref. 13). Thus, the articles reported that the demand on the emergency response system due to the dramatic increase in calls being dispatched for heat-related illnesses was compounded by a rise in accidental overdoses requiring emergency response, further taxing the already overloaded pre-hospital health system.

The fire service was also reported to have experienced increased demand due to the intersection of the overdose crisis and the 2021 Heat Dome. Specifically, Vancouver Fire and Rescue Services reported attending in one day to *“365 calls*, *including cardiac emergencies*, *heat emergencies and overdoses*,*”* which represented a *“call volume spike [of] three-fold compared to earlier [in] 2021”* (Ref. 14). The B.C. Coroner’s Service also saw similar impacts, illustrated by one article sharing that *“Chief Coroner Lisa Lapointe confirmed a ‘sharp’ increase in reported deaths have been driven by the opioid crisis and the summer ‘heat dome’ event […]*, *which she said has ‘challenged existing resources’”* (Ref. 15). This increased demand for services was also reported to have impacted the coroner’s staff. For example, it was reported that *“the tally*, *four times the average number of fatalities*, *overwhelm[ed] coroners already overburdened by the toxic drug crisis…we are experiencing stress among the coroners*, *probably more than we’ve ever seen"* (Ref. 16).

### Theme III: The barriers and stigma reported to influenc*e* an individual*’s* access to or use of heat mitigation behaviours and services

The news media articles reported that some individuals that engage in toxic drug use do not have access to air conditioning, the use of fans, and other heat mitigation options. The captured news media articles highlight that social stigma compounds these barriers to cooling. For example, one individual with lived experience shared: “*I’m a drug addict*. *It’s hard going to just any old place because people are judgmental…I’d prefer to be outside* . . .*somewhere with big shady trees”* (Ref. 17). This example emphasizes that, regardless of the availability of community cooling centres, individuals may not access them if they feel a sense of judgement from staff or other community members. Similarly, this message was reiterated in an article where a community governance group reviewed their area’s Extreme Weather Heat Response Plan: *“[Burnaby City Council] learned that homeless people cope with unusual heat with minimal protection*, *often have limited access to drinking water*, *and may face exclusion due to stigma from cooling off in air-conditioned spaces such as malls and other publicly accessible facilities”* (Ref. 18). Although this example also refers to people experiencing homelessness, given the overlap in community members that are simultaneously experiencing homelessness and using toxic drugs, it is an important consideration to extend to both populations.

### Theme IV: The reported impact of extreme heat on the public health response to drug poisoning emergencies

As a result of the 2021 Heat Dome, many community-level public health services that provide support services to individuals that use toxic drugs were reported to have been impacted. A significant aspect of these services is providing naloxone kits to temporarily reverse an opioid overdose (also known as opioid-induced respiratory depression). During the 2021 Heat Dome, *“many pharmacies received calls about drugs [i*.*e*., *Naloxone–an opioid antagonist] getting spoiled during the heat…[as] naloxone kits are all required to be stored between 15°C and 25°C”* (Ref. 19). Liquid medications like Naloxone are *“less effective when stored above recommended temperatures and may interfere with the drugs’ function”* (Ref. 20). However, during an EHE, maintaining a regular room temperature may not be possible, especially in insecure housing establishments where individuals may not have access to air conditioning or the ability to monitor the temperature of their environment. Representatives from safe consumption sites and overdose prevention centres were quoted emphasizing that *“the combined threats have [them] scrambling to get ready [at their] drop-in overdose prevention site”* (Ref. 21). Often, these references described the safe consumption sites needing to shift the focus from regular programming to the provision of water, electrolyte replacement beverages, and sun-protective supplies. For example, a representative from a drop-in overdose prevention site in downtown Prince George was quoted saying, *“The combined threats have [us] scrambling to get ready*. *We have a mountain of Gatorade*, *we have lots of sunscreen*.*”*

## Discussion

We systematically reviewed and thematically analyzed online news media articles related to toxic drug use, overdose-related deaths, and the 2021 Heat Dome in Canada. Our news media-based dataset generated four novel findings. First, toxic drug use was reported by the news media to have an impact on human thermoregulation and a person’s ability to perceive the threat posed by heat and take action to mitigate the risk posed. Second, the combined impacts of toxic drug use, overdose-related deaths, and the 2021 Heat Dome were reported to have placed an unprecedented demand on emergency healthcare services. Third, barriers and stigma surrounding individuals who use toxic drugs were reported to have influenced access to heat mitigation services. Fourth, the public health response to drug poisoning emergencies was reported to have been impacted by the extreme heat.

Based on this study’s dataset of online news media articles mentioning toxic drug use and the 2021 Heat Dome, our analysis revealed that the most prominent theme reported on was the impact of drugs on the body’s physiological capacity to thermoregulate and the individual’s ability to adopt heat mitigation behaviours. This inclusion and prioritization of risk information and warnings of the significant impact to thermoregulation aligns with the literature on the increased risk of heat-related morbidity and mortality experienced by individuals who use toxic drugs (not under the care or provision of a physician or healthcare provider). As was communicated in the media articles, the impact of toxic drug use on thermoregulation is complex and can vary depending on the specific drug (or drugs) involved. Certain toxic drugs may disrupt the normal functioning of the central nervous system, affecting the body’s ability to regulate temperature through mechanisms such as sweating and blood flow, and exacerbated by progressive dehydration caused by suppression of thirst [16]. Additionally, some toxic drugs can alter the perception of risk, leading individuals to neglect essential cooling strategies or expose themselves to extreme temperatures [17]. For example, a recent study explored three clinical case studies to demonstrate the interaction between opioid overdoses and heat exposure during an EHE in the United States [3]. In two cases, the patients used opioids in dangerously hot settings, resulting in hyperthermia, burns from touching hot surfaces inside their car, loss of consciousness, and, in one case, death. Another patient had an extended hospital course prolonged by preventable heat-related medical complications secondary to the sedative effects of opioid use. These case studies illustrate the vulnerability of individuals who use substances to heat-related illnesses due to the sedating effects of some substances, especially in settings that would place them at risk for experiencing a greater heat burden. Taken together with our findings that news media coverage is being used to raise awareness about the interaction between drug use and heat exposure, we find this to be a positive indicator for public health risk communication. However, continued work to include this information within media-based communications, perhaps with even greater coverage due to the fairly low overall prevalence within the broader dataset, could be beneficial during extreme heat events.

The reported strain posed by the intersecting crises on the health system was another prominent theme captured through our analysis of online news media articles. One of our key observations pertained to how online news media articles detailed the effects of the coinciding crises on distinct parts of the health system. This included pre-hospital emergency services (e.g., paramedic services, dispatch, fire service, and police), hospital services, and the coroner’s service. Many of the news media articles noted that excess demand placed on the emergency services was due to the increase in calls being dispatched for heat-related illnesses, which were compounded by the rise in accidental overdoses. This information could help inform public health policy, emergency response planning, and resource allocation in developing strategies to enhance the resilience of pre-hospital emergency services, the coroner’s service, and hospital services. However, to support the application of this finding further, it would be beneficial to view these findings in combination with other post-event analysis methods that capture the finalized quantifiable health records of the event (e.g., hospitalizations, emergency dispatches). Further, prioritizing news media coverage around reported impacts on the health system may indicate ‘newsworthiness’ [18]. Other studies have similarly found that coverage of extreme heat events and climate change traditionally focuses on record-breaking temperatures and impacts on critical infrastructure and services [10, 19]. This has been proposed to be due to these impacts being more in line with *news values*, which refers to criteria that influence the selection and presentation of events as published news [19]. While it is important to inform the public about the strain on the health system, particularly during an EHE, it may also be important to prioritize the dissemination of information regarding heat mitigation behaviours, especially for those that could be implemented by or in support of heat-vulnerable populations, such as individuals who use toxic drugs [20].

Our analysis of online news media articles reporting on toxic drug use and the 2021 Heat Dome also highlighted critical barriers that could have impacted an individual’s access to life-saving interventions to mitigate heat-related harms such as personal cooling options or use of community-driven heat-alleviating locations (e.g., cooling centers, air-conditioned public transit). For example, it was reported that many cooling centers do not allow toxic drug use and do not offer overdose prevention services or harm reduction services. This aligns with previous findings, which show that there is a real and perceived self-, social- and structural stigma surrounding toxic drug use that creates additional barriers to accessing community-driven cooling options [21–23]. For instance, a study that conducted qualitative interviews with people who inject drugs in California’s Central Valley found that these individuals often felt stigmatized in their interactions with pharmacists, first responders, and hospital staff, highlighting additional barriers to accessing and using health services [22, 23]. As such, future work may consider exploring the feasibility of providing supervised consumption services next to cooling centers or providing cooling strategies at existing supervised consumption sites. Further, news media articles directly reported on individuals facing exclusion from cooling off in other air-conditioned public spaces (e.g., malls, libraries). This is particularly important as many individuals at risk for drug toxicity (overdose) also have lower socioeconomic status, face racial or ethnic marginalization, and/or live in disadvantaged neighbourhoods, and thus access to home-based cooling strategies (e.g., air-conditioning, fans, cool showers or baths, or readily consumable water) may not be feasible [24]. This is particularly important as many individuals at risk for drug toxicity (overdose) also have lower socioeconomic status, face racial or ethnic marginalization, and/or live in disadvantaged neighbourhoods, and thus access to home-based cooling strategies (e.g., air-conditioning, fans, cool showers or baths, or readily consumable water) may not be feasible [24]. However, it is noteworthy that issues of race/ethnicity sociodemographic and geographic disparities were not evident in the news media articles analyzed. Public health authorities may consider actioning these findings to reduce barriers and stigma and increase the accessibility of cooling measures for this population.

Our analysis also found that extreme heat was reported to have impacted the public health response to toxic drug use and the overdose crisis. Within the captured news media articles, numerous community-based groups servicing this population adapted their specific services to help prioritize reducing the risk of heat-related illness (e.g., providing water, electrolyte replacement beverages, and shade). These types of transformations in service delivery are important as they likely play a protective role in safeguarding individuals from the health impacts of extreme heat by providing accessible cooling strategies and care. Notably, interventions aimed at reducing social isolation among members of the public, such as increased check-ins, have been demonstrated to be effective in reducing heat-related mortalities during EHEs [25]. However, these findings are limited to older adults and further studies are needed to determine the effectiveness of specific interventions aimed at reducing heat-health impacts among individuals who use toxic drugs in Canada. Additionally, it remains unclear whether the service-adaptation observed in our analysis was pre-planned or spontaneous adaptation to support the community’s needs in the face of the overlapping crises. Further research could help understand how community-based service providers adapted their services to integrate heat considerations. For example, one study found that people diagnosed with drug and alcohol dependence in Montreal, Canada, were most likely to obtain information on EHEs from community centers, community organizations, hospitals, and local health care workers [26]. This emphasizes the critical role of health and social services providers in reducing heat-health risks for this vulnerable population. Lastly, a few news media articles also mentioned that the effectiveness of naloxone kits, which are used to stop or temporarily reverse an opioid poisoning (also known as opioid-induced respiratory depression) were negatively impacted by high temperatures. During an EHE, maintaining a stable room temperature can be challenging, especially for individuals with insecure housing [27]. Thus, all individuals carrying or using naloxone may consider exploring solutions to ensure the effectiveness and safe storage of this life-saving medication.

### *Limitations* and future research

This study employed a thematic analysis of online news media articles published before, during, and after the 2021 Heat Dome in Canada. Specifically, we used this approach to identify the main themes communicated in the news media reporting on the intersection of toxic drug use, overdose-related deaths, and extreme heat. However, we recognize that our media-based dataset and analysis has important limitations and restrictions in scope that should be taken into consideration. First, although this study is part of a larger investigation which systematically reviewed and content-analyzed online news media coverage on the 2021 Heat Dome, we opted to focus the study only on articles from mass-media outlets and associations and agency press specifically within Canada. Thus, all international or social media-based sources were not explored. As the 2021 Heat Dome also impacted the northwestern United States, future work comparing the news media and social media reporting on the intersection of the toxic drug use, overdose-related deaths and the 2021 Heat Dome in the United States with Canada would be valuable. Further, the EHE selected for this analysis is now the most prominent EHE in Canadian history [28] and occurred during the COVID-19 pandemic, which was already straining the health system in Canada [6], and therefore was likely accompanied by more prominent news media coverage due to compounding burden. Further, although the dataset for this analysis remained small (3% of the total articles from the primary investigation), the findings may over emphasize certain components that would not be as dominant during a less historic heat event. Additionally, in looking to support or contrast the results of this study, it was evident that there is a lack of comparative data in Canada which limited the capacity for additional inquiry into some of the findings. This emphasizes the need for more data to be captured (or shared) to allow for improved heat-preparedness. This includes the need for perspectives of those with lived and living experience to ensure heat-health guidance and support services are reflective of actual need and consider barriers to equitable access. Lastly, because our analysis captured a broad range of articles from different news agencies to ensure it was comprehensive, the sources may contain potential biases as the articles reflect different reporting practices and interests in publishing on extreme heat, climate change, and drug use. As such, our findings reflect the interpretations of these sources as content generators and the findings should be interpreted in the context of these potential influences.

## Conclusion

Through a thematic analysis of online news media articles, we show that toxic drug use, overdose-related deaths, and the 2021 Heat Dome were reported to have had combined impacts on health and health systems in Canada. Four main themes were identified in our media-based dataset, including the reported impact of toxic drug use on thermoregulation and a person’s ability to perceive the threat of heat and take action to mitigate their risk, as well as the role of stigma in limiting accessing and use of heat mitigation strategies. The news media also often communicated how the intersecting crises led to increased demand and strain on the health system, and reported impacts to the public health response to drug poisoning emergencies. These findings may have important implications for addressing rising concerns over Canada’s growing number of overdose-related deaths and projected increases in heat-related deaths amidst rising global temperatures. The results of this study also illustrate the need for further research to better understand the consequences to health caused by toxic drug use during hot weather and EHEs. Such knowledge can help inform the development or refinement of support services, including the creation of heat-mitigation actions and response toolkits to protect individuals who use toxic drugs.

## Supporting information

S1 File News and grey literature database search strategies. (DOCX)

S2 File Database of all articles identified in the search. (XLSX)
