# Supplementary material for: The combined impacts of toxic drug use and the 2021 Heat Dome in Canada: A thematic analysis of online news media articles
Source: PLoS One. 2025 Jan 31;20(1):e0318229. doi: 10.1371/journal.pone.0318229 (PMC11785305; doi:10.1371/journal.pone.0318229)
Supplement: S1 File — (DOCX) [file pone.0318229.s001.docx]

# The combined impacts of toxic drug use and the 2021 Heat Dome in Canada: A thematic analysis of online news media articles

S1_File. News and grey literature database search strategies.

News Database Search Strategies

## ProQuest Canadian Major Dailies

NOFT(((heat* NEAR/3 (high* OR increas* OR extreme*)) OR (hot* NEAR/3 (weather* OR temperature* OR extreme* OR air)) OR ((high* OR extreme*) NEAR/3 temperature*) OR ((hot* OR heat*) NEAR/3 (day? OR week? OR month? OR unseason* OR environ* OR house* OR surroun* OR forecast* OR expos* OR episode* OR event* OR emergency OR emergencies OR disaster*)) OR (heat NEAR/3 (ill* OR disorder* OR disease* OR expos* OR overexpos* OR injur* OR health* OR "adverse effect*" OR "adverse event*" OR sick* OR admission* OR morbidit* OR mortalit* OR vulnerab* OR stress* OR fatigue* OR cramp* OR stroke* OR shock* OR exhaust* OR prostration* OR collaps* OR syncope* OR trauma* OR rash* OR strain* OR crisis OR crises OR "mass casualty" OR "mass casualties" OR hospital* OR death* OR dead* OR malaise*)) OR (heat NEAR/3 (warn* OR alert* OR broadcast* OR advisory OR advisories OR plan OR plans OR planning OR communicat* OR messag* OR respons* OR "public service announcement*" OR announcement*)) OR "heat wave*" OR heatwave* OR "heat dome*" OR heatdome* OR "hot spell*" OR "heat spell*" OR "heat island*" OR heatstroke* OR "sun stroke*" OR sunstroke* OR hyperthermi*) AND (canada* OR canadia* OR canadien* OR ottawa* OR "british columbia*" OR "colombie britannique*" OR vancouver* OR alberta* OR edmonton* OR calgar* OR saskatchewan* OR regina* OR saskatoon* OR manitoba* OR winnipeg* OR ontari* OR toronto* OR quebec* OR montreal* OR "new brunswick*" OR "nouveau brunswick*" OR fredericton* OR "nova scotia*" OR "nouvelle ecosse*" OR halifax* OR haligonian* OR "prince edward island*" OR "ile du prince edouard*" OR pei OR charlottetown* OR newfoundland* OR "terre neuve*" OR labrador* OR nfld OR yukon* OR whitehorse* OR "northwest territor*" OR "territoires du nord ouest*" OR nwt OR yellowknife* OR nunavut* OR iqaluit*))

Additional limits - Date: After January 01 2021

1,236 results

## EBSCO Business Source Elite

Advanced search:

|  | (((heat* N3 (high* OR increas* OR extreme*)) OR (hot* N3 (weather* OR temperature* OR extreme* OR air)) OR ((high* OR extreme*) N3 temperature*) OR ((hot* OR heat*) N3 (day? OR week? OR month? OR unseason* OR environ* OR house* OR surroun* OR forecast* OR expos* OR episode* OR event* OR emergency OR emergencies OR disaster*)) OR (heat N3 (ill* OR disorder* OR disease* OR expos* OR overexpos* OR injur* OR health* OR "adverse effect*" OR "adverse event*" OR sick* OR admission* OR morbidit* OR mortalit* OR vulnerab* OR stress* OR fatigue* OR cramp* OR stroke* OR shock* OR exhaust* OR prostration* OR collaps* OR syncope* OR trauma* OR rash* OR strain* OR crisis OR crises OR "mass casualty" OR "mass casualties" OR hospital* OR death* OR dead* OR malaise*)) OR (heat N3 (warn* OR alert* OR broadcast* OR advisory OR advisories OR plan OR plans OR planning OR communicat* OR messag* OR respons* OR "public service announcement*" OR announcement*)) OR "heat wave*" OR heatwave* OR "heat dome*" OR heatdome* OR "hot spell*" OR "heat spell*" OR "heat island*" OR heatstroke* OR "sun stroke*" OR sunstroke* OR hyperthermi*) AND (canada* OR canadia* OR canadien* OR ottawa* OR "british columbia*" OR "colombie britannique*" OR vancouver* OR alberta* OR edmonton* OR calgar* OR saskatchewan* OR regina* OR saskatoon* OR manitoba* OR winnipeg* OR ontari* OR toronto* OR quebec* OR montreal* OR "new brunswick*" OR "nouveau brunswick*" OR fredericton* OR "nova scotia*" OR "nouvelle ecosse*" OR halifax* OR haligonian* OR "prince edward island*" OR "ile du prince edouard*" OR pei OR charlottetown* OR newfoundland* OR "terre neuve*" OR labrador* OR nfld OR yukon* OR whitehorse* OR "northwest territor*" OR "territoires du nord ouest*" OR nwt OR yellowknife* OR nunavut* OR iqaluit*)) | TI title |
| --- | --- | --- |
| OR | (((heat* N3 (high* OR increas* OR extreme*)) OR (hot* N3 (weather* OR temperature* OR extreme* OR air)) OR ((high* OR extreme*) N3 temperature*) OR ((hot* OR heat*) N3 (day? OR week? OR month? OR unseason* OR environ* OR house* OR surroun* OR forecast* OR expos* OR episode* OR event* OR emergency OR emergencies OR disaster*)) OR (heat N3 (ill* OR disorder* OR disease* OR expos* OR overexpos* OR injur* OR health* OR "adverse effect*" OR "adverse event*" OR sick* OR admission* OR morbidit* OR mortalit* OR vulnerab* OR stress* OR fatigue* OR cramp* OR stroke* OR shock* OR exhaust* OR prostration* OR collaps* OR syncope* OR trauma* OR rash* OR strain* OR crisis OR crises OR "mass casualty" OR "mass casualties" OR hospital* OR death* OR dead* OR malaise*)) OR (heat N3 (warn* OR alert* OR broadcast* OR advisory OR advisories OR plan OR plans OR planning OR communicat* OR messag* OR respons* OR "public service announcement*" OR announcement*)) OR "heat wave*" OR heatwave* OR "heat dome*" OR heatdome* OR "hot spell*" OR "heat spell*" OR "heat island*" OR heatstroke* OR "sun stroke*" OR sunstroke* OR hyperthermi*) AND (canada* OR canadia* OR canadien* OR ottawa* OR "british columbia*" OR "colombie britannique*" OR vancouver* OR alberta* OR edmonton* OR calgar* OR saskatchewan* OR regina* OR saskatoon* OR manitoba* OR winnipeg* OR ontari* OR toronto* OR quebec* OR montreal* OR "new brunswick*" OR "nouveau brunswick*" OR fredericton* OR "nova scotia*" OR "nouvelle ecosse*" OR halifax* OR haligonian* OR "prince edward island*" OR "ile du prince edouard*" OR pei OR charlottetown* OR newfoundland* OR "terre neuve*" OR labrador* OR nfld OR yukon* OR whitehorse* OR "northwest territor*" OR "territoires du nord ouest*" OR nwt OR yellowknife* OR nunavut* OR iqaluit*)) | AB Abstract or Author-Supplied Abstract |
| OR | (((heat* N3 (high* OR increas* OR extreme*)) OR (hot* N3 (weather* OR temperature* OR extreme* OR air)) OR ((high* OR extreme*) N3 temperature*) OR ((hot* OR heat*) N3 (day? OR week? OR month? OR unseason* OR environ* OR house* OR surroun* OR forecast* OR expos* OR episode* OR event* OR emergency OR emergencies OR disaster*)) OR (heat N3 (ill* OR disorder* OR disease* OR expos* OR overexpos* OR injur* OR health* OR "adverse effect*" OR "adverse event*" OR sick* OR admission* OR morbidit* OR mortalit* OR vulnerab* OR stress* OR fatigue* OR cramp* OR stroke* OR shock* OR exhaust* OR prostration* OR collaps* OR syncope* OR trauma* OR rash* OR strain* OR crisis OR crises OR "mass casualty" OR "mass casualties" OR hospital* OR death* OR dead* OR malaise*)) OR (heat N3 (warn* OR alert* OR broadcast* OR advisory OR advisories OR plan OR plans OR planning OR communicat* OR messag* OR respons* OR "public service announcement*" OR announcement*)) OR "heat wave*" OR heatwave* OR "heat dome*" OR heatdome* OR "hot spell*" OR "heat spell*" OR "heat island*" OR heatstroke* OR "sun stroke*" OR sunstroke* OR hyperthermi*) AND (canada* OR canadia* OR canadien* OR ottawa* OR "british columbia*" OR "colombie britannique*" OR vancouver* OR alberta* OR edmonton* OR calgar* OR saskatchewan* OR regina* OR saskatoon* OR manitoba* OR winnipeg* OR ontari* OR toronto* OR quebec* OR montreal* OR "new brunswick*" OR "nouveau brunswick*" OR fredericton* OR "nova scotia*" OR "nouvelle ecosse*" OR halifax* OR haligonian* OR "prince edward island*" OR "ile du prince edouard*" OR pei OR charlottetown* OR newfoundland* OR "terre neuve*" OR labrador* OR nfld OR yukon* OR whitehorse* OR "northwest territor*" OR "territoires du nord ouest*" OR nwt OR yellowknife* OR nunavut* OR iqaluit*)) | SU Subject Terms |
| OR | (((heat* N3 (high* OR increas* OR extreme*)) OR (hot* N3 (weather* OR temperature* OR extreme* OR air)) OR ((high* OR extreme*) N3 temperature*) OR ((hot* OR heat*) N3 (day? OR week? OR month? OR unseason* OR environ* OR house* OR surroun* OR forecast* OR expos* OR episode* OR event* OR emergency OR emergencies OR disaster*)) OR (heat N3 (ill* OR disorder* OR disease* OR expos* OR overexpos* OR injur* OR health* OR "adverse effect*" OR "adverse event*" OR sick* OR admission* OR morbidit* OR mortalit* OR vulnerab* OR stress* OR fatigue* OR cramp* OR stroke* OR shock* OR exhaust* OR prostration* OR collaps* OR syncope* OR trauma* OR rash* OR strain* OR crisis OR crises OR "mass casualty" OR "mass casualties" OR hospital* OR death* OR dead* OR malaise*)) OR (heat N3 (warn* OR alert* OR broadcast* OR advisory OR advisories OR plan OR plans OR planning OR communicat* OR messag* OR respons* OR "public service announcement*" OR announcement*)) OR "heat wave*" OR heatwave* OR "heat dome*" OR heatdome* OR "hot spell*" OR "heat spell*" OR "heat island*" OR heatstroke* OR "sun stroke*" OR sunstroke* OR hyperthermi*) AND (canada* OR canadia* OR canadien* OR ottawa* OR "british columbia*" OR "colombie britannique*" OR vancouver* OR alberta* OR edmonton* OR calgar* OR saskatchewan* OR regina* OR saskatoon* OR manitoba* OR winnipeg* OR ontari* OR toronto* OR quebec* OR montreal* OR "new brunswick*" OR "nouveau brunswick*" OR fredericton* OR "nova scotia*" OR "nouvelle ecosse*" OR halifax* OR haligonian* OR "prince edward island*" OR "ile du prince edouard*" OR pei OR charlottetown* OR newfoundland* OR "terre neuve*" OR labrador* OR nfld OR yukon* OR whitehorse* OR "northwest territor*" OR "territoires du nord ouest*" OR nwt OR yellowknife* OR nunavut* OR iqaluit*)) | KW Author-Supplied Keywords |

Limiters - Published Date: 20210101-20221231

52 results

## NewsDesk

“heat wave” OR “heat dome” OR “extreme heat”

Custom Range: 1 June 2021 to 16 February 2022

Restricted: No (Removed 59)

5,534

## Factiva

((heat* near3 (high* or increas* or extreme*)) or (hot near3 (weather or temperature* or extreme* or air)) or ((high* or extreme*) near3 temperature*) or ((hot or heat*) near3 (unseason* or environ* or forecast*)) or (heat near3 (ill or illness* or disorder* or disease* or expos* or overexpos* or injur* or health* or "adverse effect*" or "adverse event*" or sick* or admission* or morbidit* or mortalit* or vulnerab* or stress* or fatigue* or cramp* or stroke* or shock* or exhaust* or prostration* or collaps* or syncope* or trauma* or rash* or strain* or crisis or crises or "mass casualty" or "mass casualties" or hospital* or death* or dead* or malaise*)) or (heat near3 (warn* or alert* or broadcast* or advisory or advisories or plan or plans or planning or communicat* or messag* or respons* or "public service announcement*" or announcement* or emergency or emergencies or disaster*)) or "heat wave*" or heatwave* or "heat dome*" or heatdome* or "hot spell*" or "heat spell*" or "heat island*" or heatstroke* or "sun stroke*" or sunstroke* or hyperthermi*) and (canad* or ottawa* or "british columbia*" or "colombie britannique*" or vancouver* or alberta* or edmonton* or calgar* or saskatchewan* or regina* or saskatoon* or manitoba* or winnipeg* or ontari* or toronto* or quebec* or montreal* or "new brunswick*" or "nouveau brunswick*" or fredericton* or "nova scotia*" or "nouvelle ecosse*" or halifax* or haligonian* or "prince edward island*" or "ile du prince edouard*" or pei or charlottetown* or newfoundland* or "terre neuve*" or labrador* or nfld or yukon* or whitehorse* or "northwest territor*" or "territoires du nord ouest*" or nwt or yellowknife* or nunavut* or iqaluit*)

**Limiters:** 1 June 2021 to 16 February 2022

**Duplicates**: Similar

**Source**: By Region - Canada

**Language**: English, French

**More Options**: Search for the free-text terms in: Headline and Lead Paragraph

**Exclude**: Republished News

2,688

## Eureka

Keywords in all the text: LEAD= ((heat* %3 (high* | increas* | extreme*)) | (hot %3 (weather | temperature* | extreme* | air)) | ((high* | extreme*) %3 temperature*) | ((hot | heat*) %3 (unseason* | environ* | forecast*)) | (heat %3 (ill | illness* | disorder* | disease* | expos* | overexpos* | injur* | health* | "adverse effect*" | "adverse event*" | sick* | admission* | morbidit* | mortalit* | vulnerab* | stress* | fatigue* | cramp* | stroke* | shock* | exhaust* | prostration* | collaps* | syncope* | trauma* | rash* | strain* | crisis | crises | "mass casualty" | "mass casualties" | hospital* | death* | dead* | malaise*)) | (heat %3 (warn* | alert* | broadcast* | advisory | advisories | plan | plans | planning | communicat* | messag* | respons* | "public service announcement*" | announcement* | emergency | emergencies | disaster*)) | "heat wave*" | heatwave* | "heat dome*" | heatdome* | "hot spell*" | "heat spell*" | "heat island*" | heatstroke* | "sun stroke*" | sunstroke* | hyperthermi*) & TEXT=(canad* | ottawa* | "british columbia*" | "colombie britannique*" | vancouver* | alberta* | edmonton* | calgar* | saskatchewan* | regina* | saskatoon* | manitoba* | winnipeg* | ontari* | toronto* | quebec* | montreal* | "new brunswick*" | "nouveau brunswick*" | fredericton* | "nova scotia*" | "nouvelle ecosse*" | halifax* | haligonian* | "prince edward island*" | "ile du prince edouard*" | pei | charlottetown* | newfoundland* | "terre neuve*" | labrador* | nfld | yukon* | whitehorse* | "northwest territor*" | "territoires du nord ouest*" | nwt | yellowknife* | nunavut* | iqaluit*))

**Date Range**: 1 June 2021 to 16 February 2022

**Search Domain**: Presse Imprimée Canada (ANG-FR)

1,308

# Grey Literature Search Strategy

In consultation with the experts on the Advisory Committee, a list of targeted websites was created for each province, including agencies related to health, the environment, agriculture, infrastructure, housing, labour, safety, hydro, along with school boards, municipalities, and Indigenous communities. Based on the geographic impact of heat dome, priority focus for detailed searching was on the western provinces (British Columbia, Alberta, Saskatchewan and Manitoba), however all 13-provinces and territories were included. For each targeted website (n=997) the terms “heat” and “2021” were entered into the search function. If no search function was available, then the researchers (ET & NG) performed a general site search targeting homepages, news tabs, newsletters, and publication/resource tabs. In addition to the targeted sites, a google search was performed for each province/territory (see search strings below). The google searches were completed until the display notice: “in order to show you the most relevant results, we have omitted some entries very similar to the X already displayed” was reached.

| **Province/Territory** | **Search String** | **Additional Filters** | **Total Results** | **Total Results Screened** | **All results prior to removing duplicates** |
| --- | --- | --- | --- | --- | --- |
| Canada | **Targeted Sites**: “heat” and “2021” | - | 15 | 416 | 7 |
|  | **Google**: "Canada" AND ("heat wave" OR "heat dome" OR "extreme heat") AND "2021" | **Country**: Canada  **Custom Range**: 1 June 2021 to 16 February 2022 | 1 | 56 | 13 |
| British Columbia | **Targeted Sites**: “heat” and “2021” | - | 455 | 8311 | 479 |
|  | **Google**: "British Columbia" AND ("heat wave" OR "heat dome" OR "extreme heat") AND "2021" | **Country**: Canada  **Custom Range**: 1 June 2021 to 16 February 2022 | 1 | 68 | 41 |
| Alberta | **Targeted Sites**: “heat” and “2021” | **-** | 381 | 10,964 | 127 |
|  | **Google**: "Alberta" AND ("heat wave" OR "heat dome" OR "extreme heat") AND "2021" | **Country**: Canada  **Custom Range**: 1 June 2021 to 16 February 2022 | 1 | 60 | 33 |
| Saskatchewan | **Targeted Sites**: “heat” and “2021” | **-** | 37 | 2,922 | 94 |
|  | **Google**: "Saskatchewan" AND ("heat wave" OR "heat dome" OR "extreme heat") AND "2021" | **Country**: Canada  **Custom Range**: 1 June 2021 to 16 February 2022 | 1 | 69 | 34 |
| Manitoba | **Targeted Sites**: “heat” and “2021” | **-** | 23 | 4008 | 73 |
|  | **Google**: "Manitoba" AND ("heat wave" OR "heat dome" OR "extreme heat") AND "2021" | **Country**: Canada  **Custom Range**: 1 June 2021 to 16 February 2022 | 1 | 58 | 38 |
| Ontario | **Targeted Sites**: “heat” and “2021” | **-** | 54 | 8265 | 78 |
|  | **Google**: "Ontario" AND ("heat wave" OR "heat dome" OR "extreme heat") AND "2021" | **Country**: Canada  **Custom Range**: 1 June 2021 to 16 February 2022 | 1 | 56 | 37 |
| Quebec | **Targeted Sites**: “heat” and “2021” | **-** | 14 | 182 | 6 |
|  | **Google**: "Quebec" AND ("heat wave" OR "heat dome" OR "extreme heat" OR “canicule” OR “vague de chaleur” OR “dome de chaleur”) AND "2021" | **Country**: Canada  **Custom Range**: 1 June 2021 to 16 February 2022 | 1 | 37 | 48 |
| New Brunswick | **Targeted Sites**: “heat” and “2021” | **-** | 12 | 101 | 5 |
|  | **Google**: "New Brunswick" AND ("heat wave" OR "heat dome" OR "extreme heat") AND "2021" | **Country**: Canada  **Custom Range**: 1 June 2021 to 16 February 2022 | 1 | 29 | 33 |
| Nova Scotia | **Targeted Sites**: “heat” and “2021” | **-** | 10 | 42 | 3 |
|  | **Google**: "Nova Scotia" AND ("heat wave" OR "heat dome" OR "extreme heat") AND "2021" | **Country**: Canada  **Custom Range**: 1 June 2021 to 16 February 2022 | 1 | 71 | 30 |
| Prince Edward Island | **Targeted Sites**: “heat” and “2021” | **-** | 8 | 197 | 4 |
|  | **Google**: "Prince Edward Island" AND ("heat wave" OR "heat dome" OR "extreme heat") AND "2021" | **Country**: Canada  **Custom Range**: 1 June 2021 to 16 February 2022 | 1 | 95 | 45 |
| Newfoundland | **Targeted Sites**: “heat” and “2021” | **-** | 12 | 194 | 0 |
|  | **Google**: "Newfoundland" AND ("heat wave" OR "heat dome" OR "extreme heat") AND "2021" | **Country**: Canada  **Custom Range**: 1 June 2021 to 16 February 2022 | 1 | 110 | 88 |
| Yukon | **Targeted Sites**: “heat” and “2021” | **-** | 8 | 3423 | 0 |
|  | **Google**: "Yukon" AND ("heat wave" OR "heat dome" OR "extreme heat") AND "2021" | **Country**: Canada  **Custom Range**: 1 June 2021 to 16 February 2022 | 1 | 102 | 55 |
| Northwest Territories | **Targeted Sites**: “heat” and “2021” | **-** | 16 | 101066 | 6 |
|  | **Google**: "Northwest Territories" AND ("heat wave" OR "heat dome" OR "extreme heat") AND "2021" | **Country**: Canada  **Custom Range**: 1 June 2021 to 16 February 2022 | 1 | 101 | 15 |
| Nunavut | **Targeted Sites**: “heat” and “2021” | **-** | 5 | 3 | 0 |
|  | **Google**: "Nunavut" AND ("heat wave" OR "heat dome" OR "extreme heat") AND "2021" | **Country**: Canada  **Custom Range**: 1 June 2021 to 16 February 2022 | 1 | 101 | 29 |
|  |  |  | **1,064** | **141,107** | **1,421** |
